# Supplementary material for: Protein language models are performant in structure-free virtual screening
Source: Brief Bioinform. 2024 Sep 27;25(6):bbae480. doi: 10.1093/bib/bbae480 (PMC11427677; doi:10.1093/bib/bbae480)
Supplement: Supplementary_Material_bbae480 [file supplementary_material_bbae480.zip › Supplementary_Information_bbae480.docx]

**Supplementary Information**

**Table of Contents**

1. Supplementary Results - Model experiments

2. Supplementary Table 1

3. Supplementary Table 2

4. Supplementary Table 3

5. Supplementary Figure 1

6. Supplementary Figure 2

7. Supplementary Figure 3

8. Supplementary File 1

9. Supplementary File 2

10. Supplementary File 3

**S1 Model experiments.** In developing the model, different model configurations were trialled. The final, most successful version is described under the methods section of this work. Notably, flipping the order of ESM-2 latents input (i.e. cross-attending to downstream parts of the ESM-2 transformer before upstream) into the model resulted in worse results. Other experiments included having the ESM-2 embeddings cross-attend to the molecular graph instead, and then mean pooling over the tokens before a small feedforward layer. These resulted in similar RMSE predictions to having the molecular graph query the ESM-2 embeddings but drastically increased the computational overhead. When training with lower gradient accumulation steps of 128, certain instances of the model failed to converge. Lastly, an implicit hydrogen model was attempted, however these failed to perform as well as the explicit hydrogen models. The authors hypothesise that the addition of hydrogen atoms resulted in lesser compression of information in the form of latent space in the model, and hence culminated in better results overall.

**Supplementary Table 1. Targets in which BIND performed the best and the worst as ranked by BEDROC in CASF-2016**

| **Best performers** | **Species** | **Protein name** |
| --- | --- | --- |
| 2al5 | *Rattus norvegicus* | GluR2 |
| 4ivc | *Homo sapiens* | JAK1 |
| 2p15 | *Homo sapiens* | Estrogen receptor |
| 4gid | *Homo sapiens* | Beta secretase |
| 4ty7 | *Homo sapiens* | Factor Xla |
| 2vw5 | *Saccharomyces cerevisiae* | Hsp90 |
| 2xb8 | *Mycobacterium tuberculosis* | Dehydroquinase |
| 1u1b | *Bos taurus* | Ribonuclease A |
| 3p5o | *Homo sapiens* | Brd4 |
| 3g0w | *Rattus norvegicus* | Androgen receptor |
|  |  |  |
| **Worst performers** | **Species** | **Protein name** |
| 3gnw | Hepatitis C virus | NS5B polymerase |
| 3uex | *Bos taurus* | Bovine beta-lactoglobulin |
| 3arp | *Vibrio harveyi* | Chitinase A |
| 3uri | *Cryphonectria parasitica* | Endothiapepsin-DB5 |
| 3coy | *Mycobacterium tuberculosis* | Pantothenate synthetase |
| 2vvn | *Bacteroides thetaiotaomicron* | GH84 |
| 3zso | Human immunodeficiency virus | Integrase |
| 3ebp | *Oryctolagus cuniculus* | Glycogen phosphorylase |
| 3nw9 | *Rattus norvegicus* | Catechol-O-methyltransferase |
| 2r9w | *Escherichia coli* | AmpC beta-lactamase |

**Supplementary Table 2. Comparison of BIND with TransformerCPI2.0 on the DEKOIS 2.0 dataset**

| **Score function / model** | **EF_0.5%_ ↑** | **EF_1%_ ↑** | **EF_5%_ ↑** |
| --- | --- | --- | --- |
| BIND | 25.36 | 24.46 | 14.43 |
| Zero-shot BIND (90% protein homology sequences removed) | 16.84 | 15.19 | 9.24 |
| TransformerCPI2.0 | 6.46 | 5.49 | 3.32 |

**Supplementary Table 3. Comparison of BIND with TransformerCPI2.0 on the DUD-E dataset**

| **Score function / model** | **EF_0.5%_ ↑** | **EF_1%_ ↑** | **EF_5%_ ↑** |
| --- | --- | --- | --- |
| BIND | 51.88 | 46.35 | 15.92 |
| Zero-shot BIND (90% protein homology sequences removed) | 30.52 | 26.39 | 10.46 |
| TransformerCPI2.0 | 11.03 | 8.49 | 4.29 |


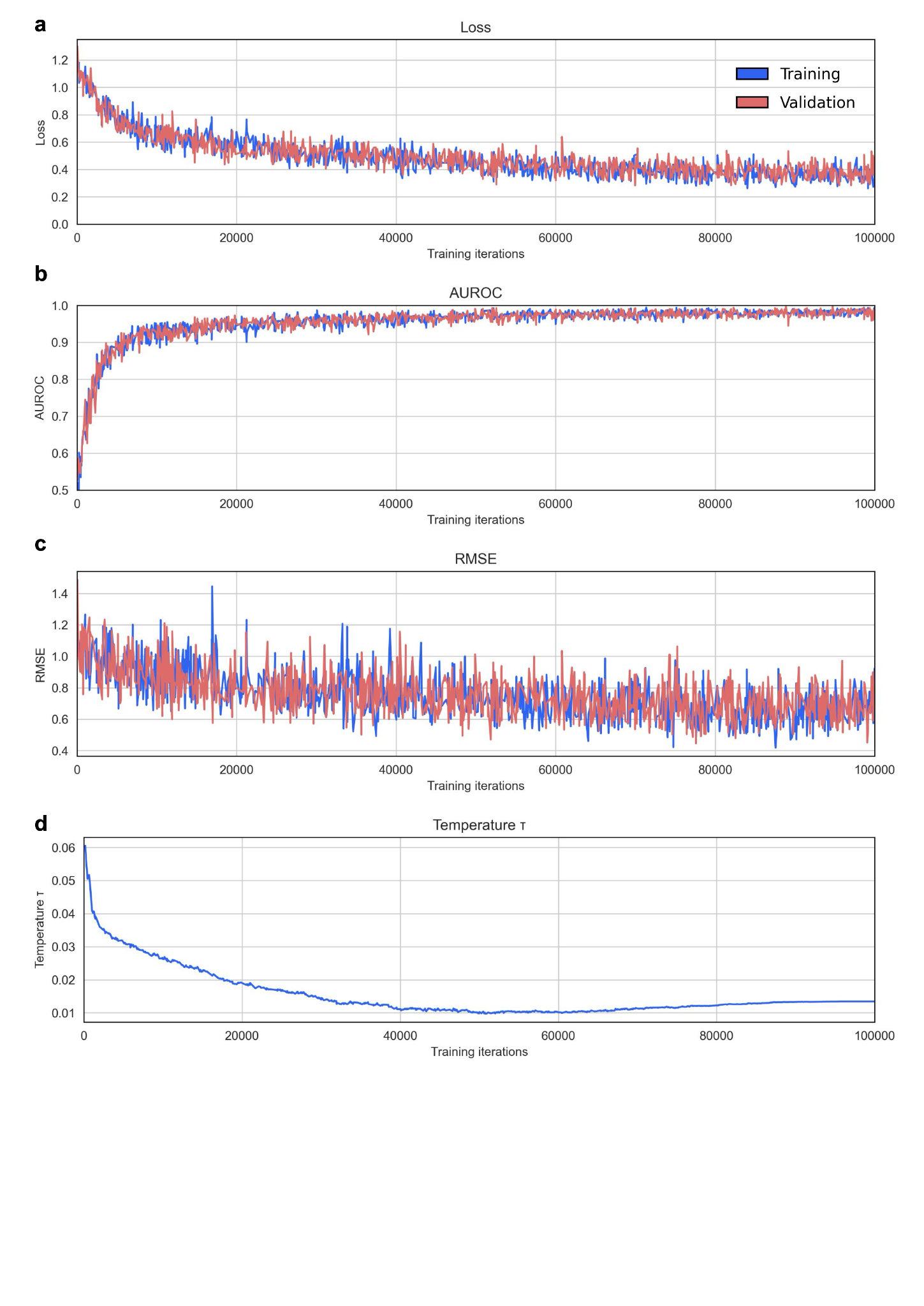


**Supplementary Figure 1. Training and evaluation metrics for the model.** (a) Loss curve with a combined loss of Huber for the DTA regression and binary cross entropy for decoy/true binder classification; (b) AUROC train and validation curve; (c) DTA RMSE train and validation curve; (d) temperature parameter throughout training.

**
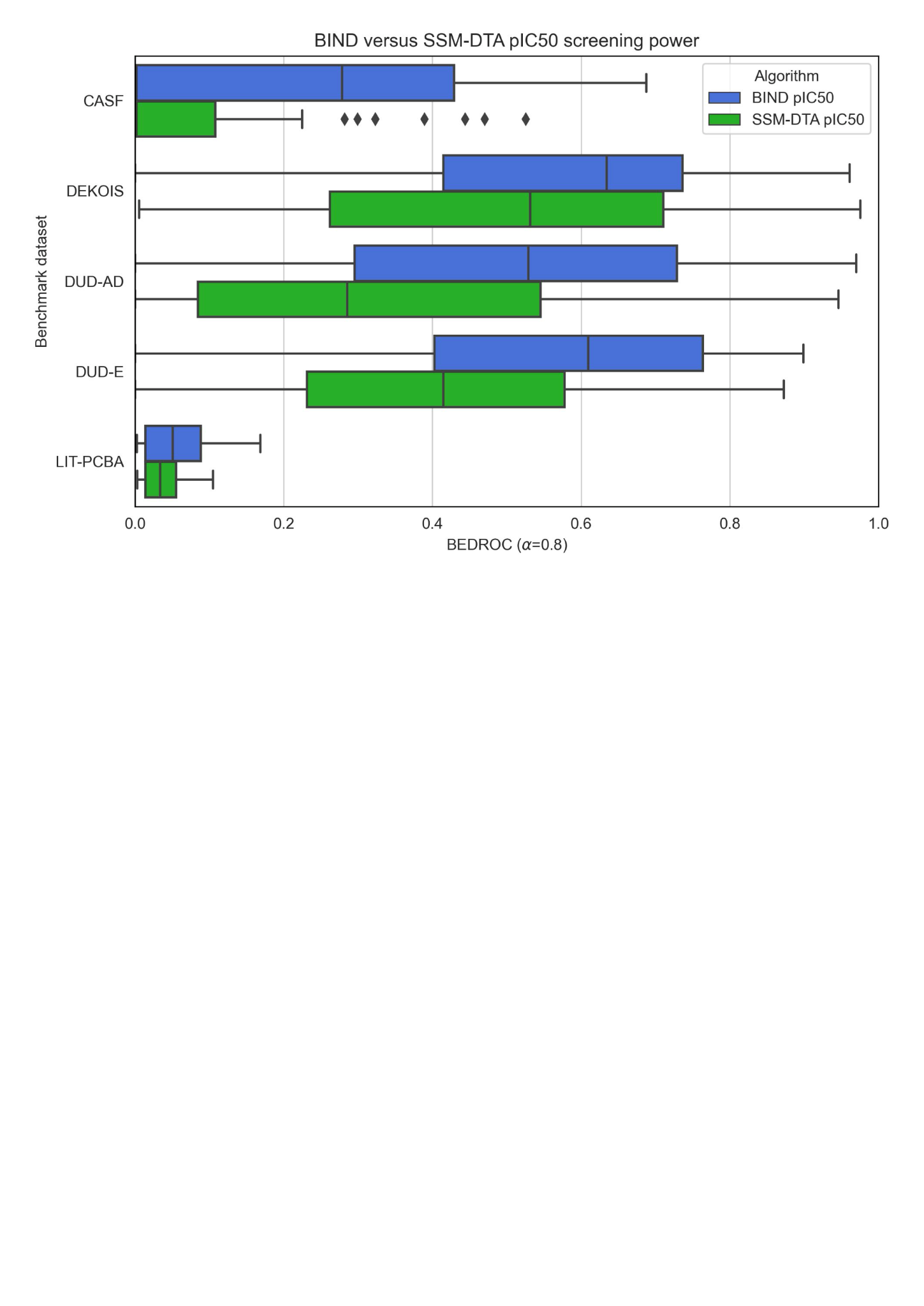
**

**Supplementary Figure 2. BIND still achieves higher screening power than SSM-DTA on its pIC_50_ prediction despite SSM-DTA’s being more accurate when measured via the RMSE metric.** The BEDROC values across the CASF-2016, DEKOIS, DUD-AD, DUD-E and LIT-PCBA datasets are compared and calculated by ranking on the pIC_50_ predictions of both BIND and SSM-DTA. Boxes represent the interquartile range with the median demarcated in the box, whiskers showing the fence and diamonds showing outliers.


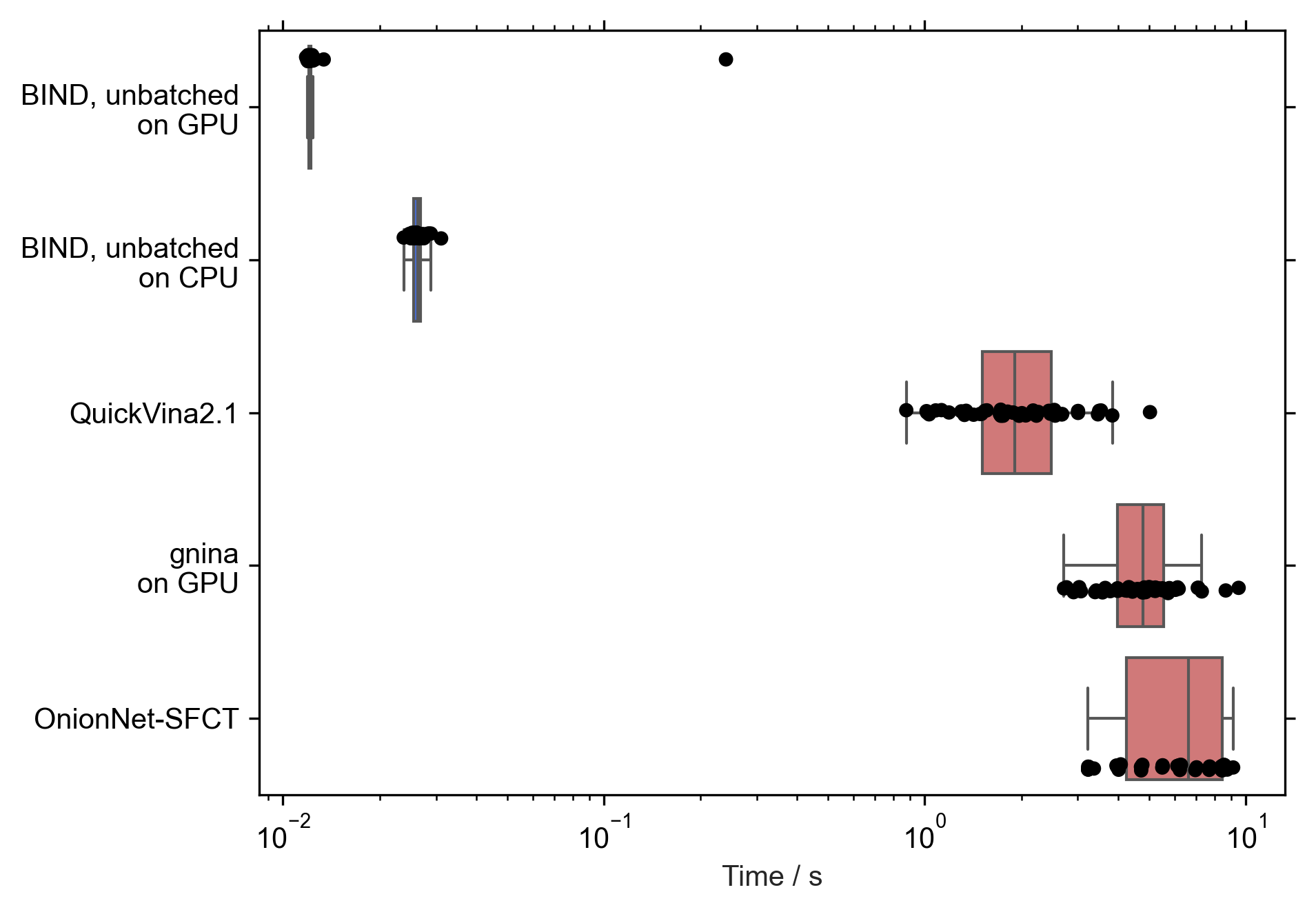


**Supplementary Figure 3. BIND is significantly faster than other SBDD models.** 50 random ligands were selected and docked against 2QFT, the EPSP synthase in *E. coli*., a 429 amino acid sequence. The time taken for BIND is calculated with ESM-2 being only run once at initialisation and not included in the time taken as one protein embedding has to be calculated per protein and it can be cached and reused for different molecules. Boxes represent the interquartile range with the median demarcated in the box, whiskers showing the fence and diamonds showing outliers. Dots indicate individual bootstraps.

**Supplementary File 1. All evaluation metrics across individual targets in different evaluation datasets using BIND.**

**Supplementary File 2. All evaluation metrics across individual targets in different evaluation datasets using zero-shot BIND.**

**Supplementary File 3. Protein sequences used in evaluation on CASF-2016, DEKOIS 2.0, DUD-AD, DUD-E, and LIT-PCBA.**
