# Supplementary material for: Protein language models are performant in structure-free virtual screening
Source: Brief Bioinform. 2024 Sep 27;25(6):bbae480. doi: 10.1093/bib/bbae480 (PMC11427677; doi:10.1093/bib/bbae480)
Supplement: Supplementary_Material_bbae480 [file supplementary_material_bbae480.zip › Supplementary_Table_1_bbae480.docx]

**Supplementary Table 1. Targets in which BIND performed the best and the worst as ranked by BEDROC in CASF-2016**

| **Best performers** | **Species** | **Protein name** |
| --- | --- | --- |
| 2al5 | *Rattus norvegicus* | GluR2 |
| 4ivc | *Homo sapiens* | JAK1 |
| 2p15 | *Homo sapiens* | Estrogen receptor |
| 4gid | *Homo sapiens* | Beta secretase |
| 4ty7 | *Homo sapiens* | Factor Xla |
| 2vw5 | *Saccharomyces cerevisiae* | Hsp90 |
| 2xb8 | *Mycobacterium tuberculosis* | Dehydroquinase |
| 1u1b | *Bos taurus* | Ribonuclease A |
| 3p5o | *Homo sapiens* | Brd4 |
| 3g0w | *Rattus norvegicus* | Androgen receptor |
|  |  |  |
| **Worst performers** | **Species** | **Protein name** |
| 3gnw | Hepatitis C virus | NS5B polymerase |
| 3uex | *Bos taurus* | Bovine beta-lactoglobulin |
| 3arp | *Vibrio harveyi* | Chitinase A |
| 3uri | *Cryphonectria parasitica* | Endothiapepsin-DB5 |
| 3coy | *Mycobacterium tuberculosis* | Pantothenate synthetase |
| 2vvn | *Bacteroides thetaiotaomicron* | GH84 |
| 3zso | Human immunodeficiency virus | Integrase |
| 3ebp | *Oryctolagus cuniculus* | Glycogen phosphorylase |
| 3nw9 | *Rattus norvegicus* | Catechol-O-methyltransferase |
| 2r9w | *Escherichia coli* | AmpC beta-lactamase |
