# Supplementary material for: Protein language models are performant in structure-free virtual screening
Source: Brief Bioinform. 2024 Sep 27;25(6):bbae480. doi: 10.1093/bib/bbae480 (PMC11427677; doi:10.1093/bib/bbae480)
Supplement: Supplementary_Material_bbae480 [file supplementary_material_bbae480.zip › Supplementary_Table_2_bbae480.docx]

**Supplementary Table 2. Comparison of BIND with TransformerCPI2.0 on the DEKOIS 2.0 dataset**

| **Score function / model** | **EF_0.5%_ ↑** | **EF_1%_ ↑** | **EF_5%_ ↑** |
| --- | --- | --- | --- |
| BIND | 25.36 | 24.46 | 14.43 |
| Zero-shot BIND (90% protein homology sequences removed) | 16.84 | 15.19 | 9.24 |
| TransformerCPI2.0 | 6.46 | 5.49 | 3.32 |
