# Supplementary material for: Protein language models are performant in structure-free virtual screening
Source: Brief Bioinform. 2024 Sep 27;25(6):bbae480. doi: 10.1093/bib/bbae480 (PMC11427677; doi:10.1093/bib/bbae480)
Supplement: Supplementary_Material_bbae480 [file supplementary_material_bbae480.zip › Supplementary_Table_3_bbae480.docx]

**Supplementary Table 3. Comparison of BIND with TransformerCPI2.0 on the DUD-E dataset**

| **Score function / model** | **EF_0.5%_ ↑** | **EF_1%_ ↑** | **EF_5%_ ↑** |
| --- | --- | --- | --- |
| BIND | 51.88 | 46.35 | 15.92 |
| Zero-shot BIND (90% protein homology sequences removed) | 30.52 | 26.39 | 10.46 |
| TransformerCPI2.0 | 11.03 | 8.49 | 4.29 |
