# Supplementary figures and images for: Protein language models are performant in structure-free virtual screening
Source: Brief Bioinform. 2024 Sep 27;25(6):bbae480. doi: 10.1093/bib/bbae480 (PMC11427677; doi:10.1093/bib/bbae480)

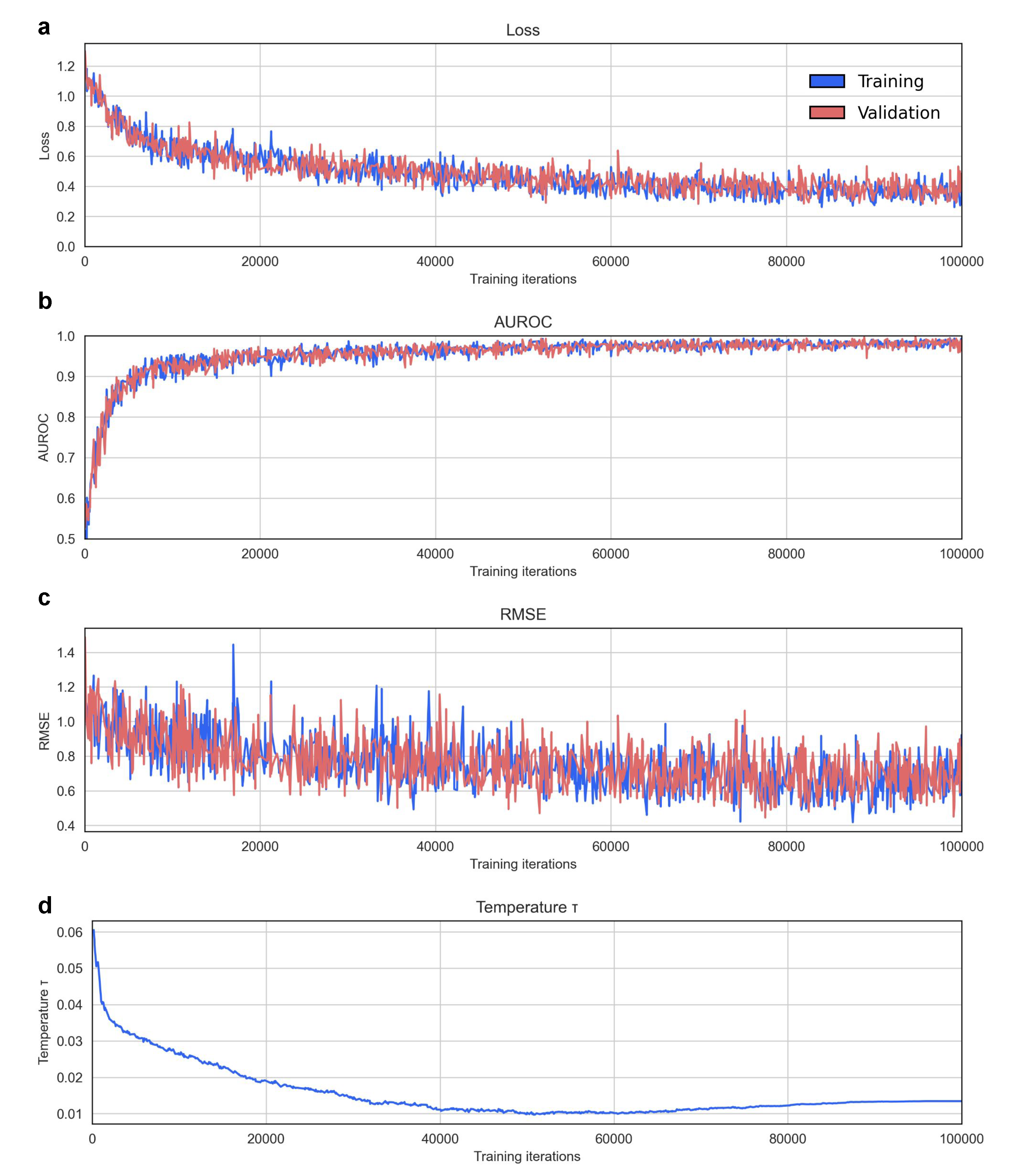

Supplement: Supplementary_Material_bbae480 [file supplementary_material_bbae480.zip › Supplementary_Figure_1_bbae480.png]

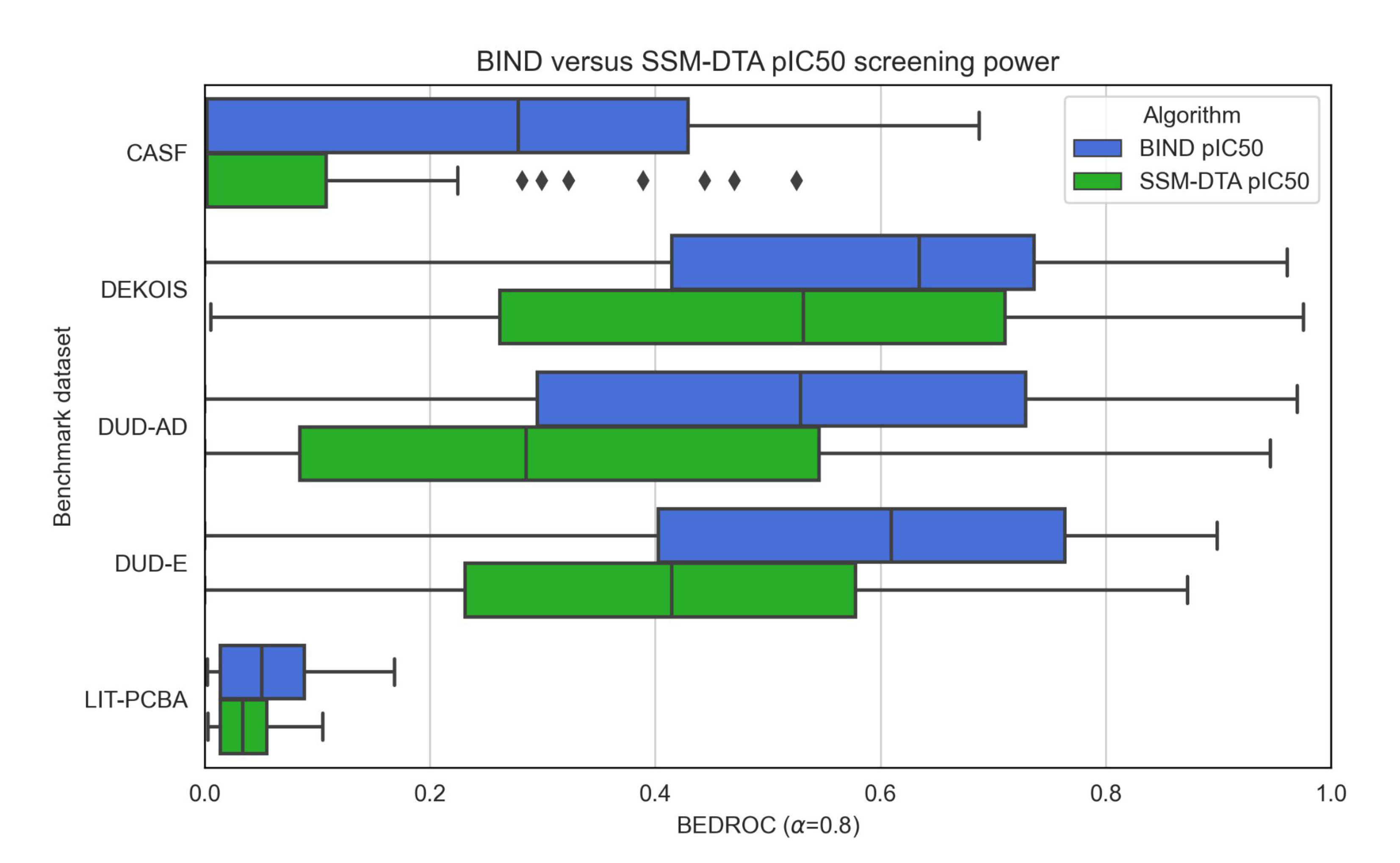

Supplement: Supplementary_Material_bbae480 [file supplementary_material_bbae480.zip › Supplementary_Figure_2_bbae480.png]

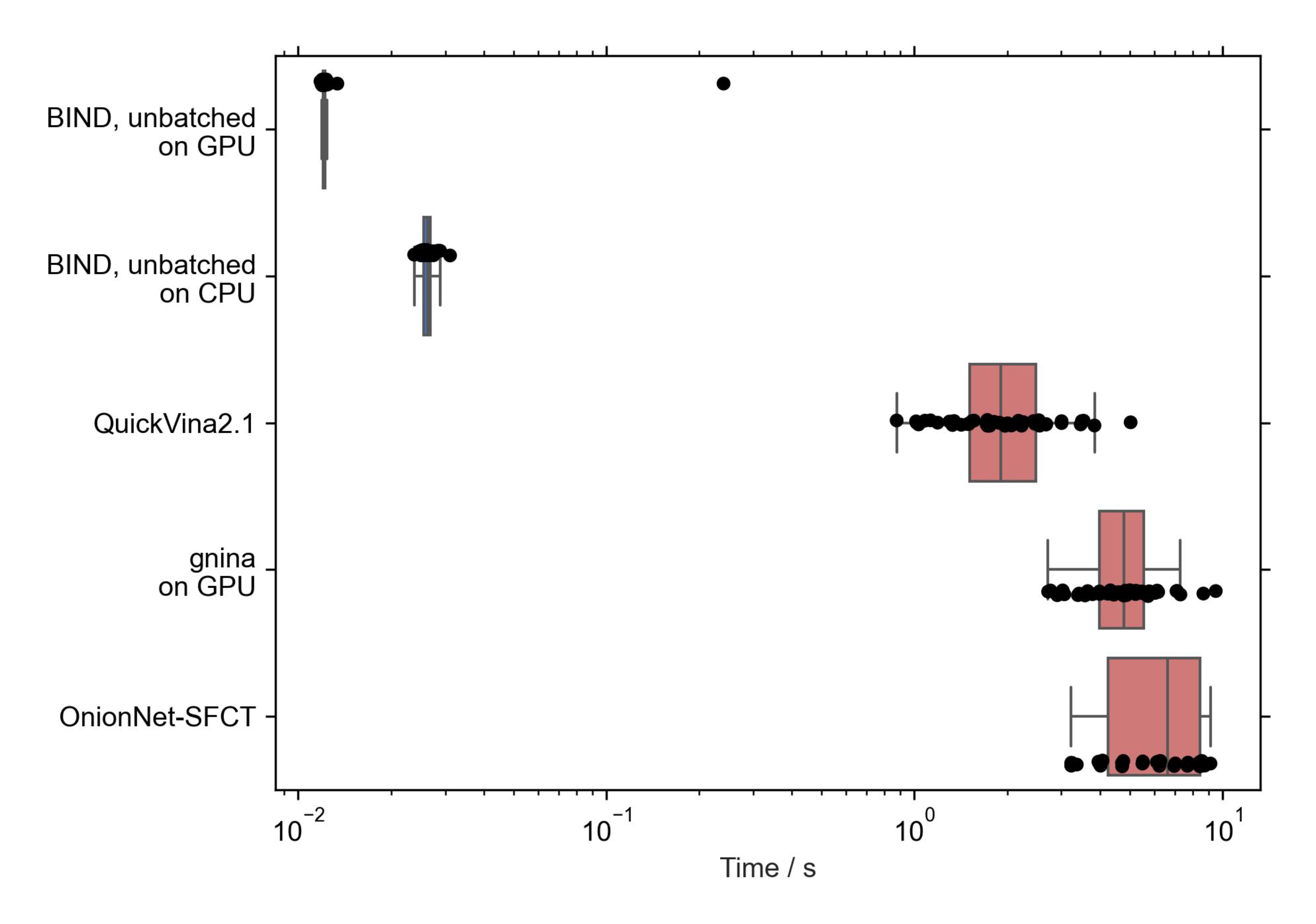

Supplement: Supplementary_Material_bbae480 [file supplementary_material_bbae480.zip › Supplementary_Figure_3_bbae480.png]
